# IPF-Acute Exacerbations: Advances and Future Perspectives

**DOI:** 10.3389/fphar.2022.836553

**Published:** 2022-04-14

**Authors:** Spyros A. Papiris, Lykourgos Kolilekas, Konstantinos Kagouridis, Maria Maniati, Effrosyni D. Manali

**Affiliations:** ^1^ 2ndPulmonary Medicine Department, General University Hospital “Attikon”, Medical School, National and Kapodistrian University of Athens, Athens, Greece; ^2^ 7th Pulmonary Department, Athens Chest Hospital “Sotiria”, Athens, Greece

**Keywords:** idiopathic pulmonary fibrosis, acute exacerbations, acute respiratory distress syndrome, microbiome, infections, steroids, immunosuppressants

## Introduction

Idiopathic pulmonary fibrosis (IPF) is the “mother” of all idiopathic fibrotic interstitial lung diseases (ILDs), the most common and clinically severe, and although it invariably presents an ominous prognosis, its clinical course is highly unpredictable, lasting at diagnosis from months to almost a decade (Kim et al., 2015; Raghu et al., 2015; Raghu et al., 2018). Acute deteriorations during its clinical course, fulfilling the criteria for the development of acute respiratory distress syndrome (ARDS), the so-called IPF acute exacerbations (AEs), represent the most devastating of its complications and, if untreated, lead to death in almost all patients in the time space of few days (Collard et al., 2016). Avoiding the ICU in these patients because of their high mortality is an option, and independently of any treatment or support, more than 40% of all deaths in IPF relate to an AE event (Natsuizaka et al., 2014).

## Historical Perspective

Although Hamman and Rich are considered the first to describe, in the early thirties, the new diffuse interstitial fibrotic lung clinical entity, presumably IPF (Hamman and Rich, 1935), its first description probably antedates to 1872 when von Buhl, in the German-language literature, reported it under the name of desquamative pneumonia, a histological entity that resembles the usual interstitial pneumonia (UIP) histology of IPF (Von Buhl, 1872). Before Hamman and Rich, other German-language authors, namely, Rindfleisch a case report, 1898 (Rindfleisch, 1898); Sandoz the first familial IPF, 1907 (Sandoz, 1907); and von Hansemann a case series 1912 (Von Hansemann, 1912), reported probably what we name today IPF. However, they were without any evidence. Hamman and Rich were the first to describe an acutely (fulminant) deteriorated IPF since their clinical recognition of “an acute diffuse interstitial fibrosis of the lungs” and their detailed histologic description “… alveolar edema … hyaline membrane formation … cuboidal proliferation of alveolar cells … leukocytes … excessive proliferation of fibrous tissue in the interstitium … necrosis of alveolar and bronchiolar walls” (Hamman and Rich, 1935) resembles the diffuse alveolar damage (DAD) histology on the UIP pattern of an ARDS development upon IPF, previously reported as IPF-AEs.

### The Modern History

The modern history of IPF-AEs begins probably in 1993 when Yashuhiro Kondoh reported a case series of three patients with IPF fulfilling ARDS criteria including PaO_2_/FiO_2_ ≤ 300, presenting with “influenza-like symptoms and/or cough and fever; leukocytosis and high CRP; with biopsy proven, at least in two of them; and UIP histology and acute lung injury (ALI)” (Kondoh et al., 1993). The “abundance of fibroblastic foci” in at least two of them led the authors to speculate and suggest that the aforementioned clinical context might represent “a fulminant active state of UIP and may be considered one morphologic expression of the acute exacerbation” (Kondoh et al., 1993). Since at that time the standard care in stable IPF patients was the administration of steroids and azathioprine or cyclophosphamide, Kondoh et al. opted for the multiplication of the dose of steroids in the form of steroid pulse administration and referred improvement (Kondoh et al., 1993).

### The First Expert’s Report

Thirteen years later, in an international expert’s report on IPF, the idiopathic nature of IPF-AEs constitutes the first hypothesis on their etiology, though the dilemma “do acute exacerbations of IPF represent a distinct pathobiological manifestation of the primary disease process or are they caused by occult complications such as infection and aspiration” dominates the key questions remaining for future research (Collard et al., 2007). In the same document, it is recognized that “diffuse alveolar damage superimposed on underlying UIP is the most commonly described finding when surgical lung biopsy is performed in patients with AE-IPF” (Kondoh et al., 1993; Ambrosini et al., 2003; Rice et al., 2003; Parambil et al., 2005; Kim et al., 2006), although “organizing pneumonia without other evidence of organizing diffuse alveolar damage and extensive fibroblastic foci” has also been described in a few cases (Churg et al., 2007) and that “treatment of IPF-AEs has generally consisted of high-dose corticosteroids, without any data from controlled trials to prove their efficacy.” Admission in some cases equals permission for most, and the aforementioned therapeutic approach represents the standard of care to date all over the world in both specialized and non-specialized ILD centers (Polke et al., 2021). However, in most centers, broad-spectrum antibiotics plus macrolides were also administered and in some of them, immunosuppressants too were administered (cyclosporine A, intravenous cyclophosphamide, or tacrolimus) (Polke et al., 2021).

### The Rising of an Alternative Thinking

However, the aforementioned considerations regarding theory and practice of IPF-AEs were not universally adopted, and our group of investigators in a review article based on own clinical observation sustained that in the clinical context of a rapidly deteriorating IPF patient, clinicians are faced with three different scenarios: first, the progression toward the “final end” of the disease where spontaneous breathing becomes unsupportable because of the excessive fibrotic lung derangement and where palliation of breathlessness appears the only option; second, the development of a new clinical complication (infection, embolism, or heart decompensation), reversible when promptly diagnosed and appropriately treated; and third, the true development of ARDS upon IPF (Papiris et al., 2010). In this clinical context and since in the etiology of ARDS, pneumonia and sepsis are the most commonly encountered triggering factors, and in those years, most patients were receiving immunosuppressants that clearly predisposed them to infections; the authors suggested the withdrawal of the aforementioned immunosuppressants and treatment according to the ARDS guidelines: provision of excellent supportive care, any effort to identify and treat triggering factors, and in the process of identification or if unidentified, administration of antibiotics according to the immunological status and clinical context of the patient (Ranieri et al., 2012). Therefore, not all clinical deteriorations in IPF patients constitute AEs and naturally, all clinicians are aware of the difficult prognosis of ARDS developing in fibrosis-deranged lungs such as those of IPF patients (Bellani et al., 2016). The suggested approach by the authors aimed to avoid the “paradox” in IPF-AE treatment of increasing immunosuppression in the already immunosuppressed patient developing ARDS, advancing also the possibility to increase survivors. The next year, “evidence-based guidelines for diagnosis and treatment of IPF” insisted on steroid “pulse” treatment for IPF-AEs (Raghu et al., 2011), which was promptly argued as inappropriate and not “evidenced” by our group (Papiris et al., 2012).

### The Real Practice

Soon after, the results of the seminal study for IPF “the PANTHER trial” by Raghu et al. were announced and published, showing that the triple treatment, prednisone–azathioprine–*N-*acetylcysteine versus placebo “was associated with increased all-cause mortality, all-cause hospitalizations, and treatment-related severe adverse events; deaths and hospitalizations happened early in the combination-therapy group,” and finally, AEs developed only in the treatment arm of the study (Raghu et al., 2012). The study definitely provided evidence not only against the use of the aforementioned treatment for stable IPF but also for the first-time evidence on the potential deleterious role of immunosuppressants regarding the development of IPF-AEs (Raghu et al., 2012). A few years later, another study by our group showed that a history of immunosuppression before the development of IPF-AE has a negative impact on its outcome by increasing mortality (Papiris et al., 2015). From both the aforementioned clinical studies, the putative role of immunosuppressants as triggers of infections in IPF-AEs appears to acquire consistency.

### The Time of Discovery of Lung Microbiome

The time has come from research findings on the lung “microbiome” to challenge the traditional knowledge that the human alveolar space is sterile (Lederberg, 2001; Dickson et al., 2014). Using molecular culture–independent techniques, it was Hilty et al. who documented first in 2010 that “the bronchial tree contains a characteristic microbiota” in the healthy state and suggested that this microbiota is negatively influenced in diseases such as bronchial asthma (Hilty et al., 2010). The microbiome of the healthy respiratory tract has been extensively studied, found present even during prenatal lung development (Dickson et al., 2016). Chronic lung diseases such as cystic fibrosis, COPD, bronchial asthma, bronchiectasis, and IPF contain a dysbiotic microbiome (Dickson et al., 2016). Therapies for chronic lung diseases including inhaled and systemic corticosteroids also negatively affect the microbiome (Dickson et al., 2016). In IPF, the COMET study showed for the first time that the lung microbiome is related to disease progression and poor outcome (Han et al., 2014). Molyneaux et al. further advanced this knowledge by showing that stable IPF patients have high bacterial burden which further increases at exacerbation and changes in composition (Molyneaux et al., 2014; Molyneaux et al., 2017a). Accordingly, Weng et al. found that patients with IPF developing AEs have 38 different bacterial strains in their sputum (Weng et al., 20192019), while Xue et al. found colonization with *Pneumocystis jirovecii* in a significant number of patients with stable IPF (Xue et al., 2020). The lung microbiome, which differs between stable and progressive diseases, elicits a defense response by the host since it persistently and repeatedly injures the alveolar epithelia, and in both the animal model and humans, it affects the clinical outcome (Molyneaux et al., 2017a; Molyneaux et al., 2017b; O'Dwyer et al., 2019; Valenzi et al., 2021). In addition to the revolutionary advances in the understanding of the putative role of bacteria in the pathogenesis and progression of IPF and of their uprising role in the development of IPF-AEs, the scientific community still seems rather skeptical about recommending antibiotics according to clinical judgement and considering corticosteroids for ALI in addition to supportive measures as the mainstay of treatment in IPF-AEs (Maher et al., 2015). The evidence regards mostly stable IPF (Martinez et al., 2021), whereas randomized, controlled trials examining the role of antimicrobials in the management of IPF-AEs are still lacking. Furthermore, the role of antimicrobials is scarcely mentioned or analyzed in trials examining the immunosuppressive treatment in IPF-AE, in addition to their widespread use in everyday clinical practice (Polke et al., 2021). In a retrospective study by Oda et al., it was shown that the administration of co-trimoxazole and macrolides was significantly associated with good prognosis in ventilated patients with IPF showing a rapid progression of respiratory failure and being treated with high doses of steroids (Oda et al., 2016).

### The New International Expert Report

One step ahead, constitutes the new revised definition and diagnostic criteria for IPF-AEs, an international working-group report, which removes “idiopathic” from their definition and parallels the criteria of IPF-AEs to the Berlin criteria for ARDS (Collard et al., 2016). The report, however, borrows but not completely adopts the Berlin criteria, though the development of DAD on UIP actually called the IPF-AE event, to our opinion, substantially equals ARDS of any etiology on IPF and should be managed accordingly (Papiris et al., 2017). The next year, a study on biomarkers of the early phase of IPF-AEs evidenced high levels of IL-6 and IL-8 cytokines in ARDS and failed to identify any increase of fibroblastic growth markers (Papiris et al., 2018).

## Original Research

A retrospective study by Farrand et al. from the US on IPF-AEs added new evidence to the deleterious role of steroids on their clinical course since patients treated with steroids required more ICU stay and mechanical ventilation, presenting reduced survival (Farrand et al., 2020). Similarly, a retrospective study from Korea failed to show improved outcomes in IPF-AEs from high-dose steroid administration (Jang et al., 2021). To consolidate the knowledge on the role of steroids in the management of IPF-AEs certainly demands larger cohorts and controlled prospective studies, but the common practice of opting for their administration in high-dose pulses intending to halt the rapidly evolving clinical situation of the IPF patient under AE begins to appear less appealing, and an international debate “pro e con” steroids arises (Papiris et al., 2017; Arai and Inoue, 2018; Sellarés and Bermudo, 2018; Arai and Inoue, 2020).

The “eagerly awaited” results of the EXAFIP trial were recently published (Naccache et al., 2021). EXAFIP was a randomized placebo-controlled trial performed in 31 hospitals all over France comparing the effect of intravenous pulses of cyclophosphamide added to high-dose systemic steroids in non-mechanically ventilated patients hospitalized for IPF-AEs. The patients were receiving either intravenous cyclophosphamide on days 0, 15, 30, and 60 or the placebo. All patients were receiving high-dose steroid therapy and prophylaxis against *Pneumocystis jirovecii*. The results of the trial showed that the addition of intravenous pulses of cyclophosphamide to high-dose steroids failed to show any survival benefit in patients with IPF-AEs. The higher mortality with cyclophosphamide than with placebo suggested, instead, a deleterious effect, providing strong evidence against its use. According to the authors, respiratory insufficiency due to the progression or recurrence of an IPF-AE event was the main cause of death in both placebo-steroid and cyclophosphamide-steroid groups, and no difference was observed in the incidence of all infectious-related serious adverse events in both groups. The EXAFIP trial should be acknowledged as a trial of remarkable importance in its field not only because it dismissed the assertion of the past that cyclophosphamide could be of any benefit in the treatment of IPF-AEs but also because it added evidence on the deleterious role of steroids on IPF-AEs since the most frequent adverse event reported in the study, that of infectious disease, was developed equally (33 vs. 36%) in both arms (CYC + steroids versus steroids alone). However, clinicians and researchers seem persuaded for the need of a new randomized controlled trial to address the efficacy and safety of steroids alone in IPF-AEs in the future (trial already named EXAFIP2) (Corticostéroïdes, 2020).

## Comments and Conclusion

Several considerations including ethical ones may arise in the designing of new studies on steroids: an arm of the placebo without antimicrobials may not be ethical and using antimicrobials in both arms is against testing the hypothesis of the idiopathic nature of IPF-AEs; but do we really need new trials? Both the PANTER and EXAFIP trials are consistent regarding their results: the PANTHER trial demonstrated that in stable IPF, the combination of steroids and immunosuppressants is associated with increased mortality and induces AEs (Raghu et al., 2012), and the EXAFIP trial demonstrated that the combination of steroids and another immunosuppressant is catastrophic in the treatment of patients who have already developed an AE (Naccache et al., 2021). The common denominator in both is steroids. Do we need more evidence about their role in both stable and exacerbated diseases to finally answer the question “steroids: defenders or killers” (Papiris et al., 2012)? The treatment of IPF-AEs still awaits an orthologous medical approach. The equal pairing of IPF-AE criteria with those of ARDS and the adoption of its therapeutic approach might be the next and probably the ultimate step.

In conclusion, for some, controversy still surrounds the treatment of IPF-AEs, but not for all (Papiris et al., 2014). “Well and appropriately designed studies” are needed, are ethical, or the time has come to attain a decision against the “paradox” of the common practice in IPF-AEs (Polke et al., 2021), long awaited and owed to our patients.
